# A decision support system (*GesCoN*) for managing fertigation in open field vegetable crops. Part I—methodological approach and description of the software

**DOI:** 10.3389/fpls.2015.00319

**Published:** 2015-05-20

**Authors:** Antonio Elia, Giulia Conversa

**Affiliations:** Department of the Science of Agriculture, Food and Environment, University of FoggiaFoggia, Italy

**Keywords:** crop growth modeling, nitrogen uptake simulation model, nitrate vulnerable zones, sustainable fertilization, nutrient budgeting

## Abstract

Reduced water availability and environmental pollution caused by nitrogen (N) losses have increased the need for rational management of irrigation and N fertilization in horticultural systems. Decision support systems (DSS) could be powerful tools to assist farmers to improve irrigation and N fertilization efficiency. Currently, fertilization by drip irrigation system (fertigation) is used for many vegetable crops around the world. The paper illustrates the theoretical basis, the methodological approach and the structure of a DSS called *GesCoN* for fertigation management in open field vegetable crops. The DSS is based on daily water and N balance, considering the water lost by evapotranspiration (ET) and the N content in the aerial part of the crop (N uptake) as subtraction and the availability of water and N in the wet soil volume most effected by roots as the positive part. For the water balance, reference ET can be estimated using the Penman–Monteith (PM) or the Priestley–Taylor and Hargreaves models, specifically calibrated under local conditions. Both single or dual Kc approach can be used to calculate crop ET. Rain runoff and deep percolation are considered to calculate the effective rainfall. The soil volume most affected by the roots, the wet soil under emitters and their interactions are modeled. Crop growth is modeled by a non-linear logistic function on the basis of thermal time, but the model takes into account thermal and water stresses and allows an in-season calibration through a dynamic adaptation of the growth rate to the specific genetic and environmental conditions. N crop demand is related to DM accumulation by the N critical curve. N mineralization from soil organic matter is daily estimated. The DSS helps users to evaluate the daily amount of water and N fertilizer that has to be applied in order to fulfill the water and N-crop requirements to achieve the maximum potential yield, while reducing the risk of nitrate outflows.

## Introduction

The need for rational management of irrigation and nitrogen (N) fertilization has become an issue for the agricultural systems around the world and in particular in Mediterranean regions as a result of reduced water availability and of the environmental pollution caused by nitrogen losses. Underground and surface water pollution from agricultural sources of nitrate (NO^−^_3_) is a problem that the European member States are facing. They must especially manage the horticultural sector, in view of achieving the sets of water quality objectives (Nitrates Directive—1991/676/EEC—and Water Framework Directive—2000/60/EC—objectives). Restrictions in N fertilization application are imposed to prevent the outflow of nitrates from agricultural sources in zones designed as nitrate vulnerable (NVZs).

Currently, fertilization by drip irrigation system (fertigation) is used for many vegetable crops around the world, mainly in Mediterranean areas. This system allows great flexibility in water and nutrition management, offering the potential to increase water and nutrient-use efficiency. However, the advantages associated with fertigation are closely linked to the supply to the root zone of the precise water volume and the N rate necessary to meet crop requirements during growth and development. Due to the high solubility of nitrates, the leaching of nitrate beyond the root zone is a potential problem associated with fertigation (Cook and Sanders, 1991; Thompson and Doerge, 1996). Therefore, careful management of N and water applications can minimize the amount of nitrates moving below the root zone, thus reducing the risk of contamination of ground waters.

Simplified Decision support systems (DSS) derived from crop growth models, simulating crop N and water requirements can be very helpful tools for farmers operating with field grown vegetable crops when determining the optimum management of irrigation and N fertilizer application through fertigation. This aspect is crucial in zones vulnerable to nitrates from agricultural sources.

Several software packages have been proposed in recent years to model water, C and N dynamics in the soil–crop system (Kersebaum et al., 2007; Rinaldi and He, 2014), but few of them have been developed with the aim of assisting irrigation or N fertilization at the farm scale. These software packages differ markedly in complexity according to the functional objective for which they were designed. Frequently they require numerous off-farm input data for both soil and crop sub-models that are not always available to farmers and most of them are mainly developed to operate with arable crops (wheat, barley, maize), in which fertigation is generally not used. In another case, where fertigation in open field conditions is considered, the quantification of crop N demand is based only on tabular data (Moreira Barradas et al., 2012). However, no software is currently available to manage fertigation in NVZs.

The hypothesis behind this work was to create an efficient, easy-to-use, flexible and adaptive decision support tool intended to manage fertigation at the field scale in open field grown vegetable crops.

The present paper illustrates the theoretical basis, the methodological approach, the main algorithms used and contains a description of the model operation of this DSS software program—called *GesCoN*.

With the aim of providing accurate daily recommendations, *GesCoN* uses the daily water and nitrogen balance at the field scale by modeling (a) crop growth and the related N uptake, through a dynamic approach, also taking into account water and thermal stresses, (b) crop water requirements though daily ET_0_ estimation using the Penman–Monteith equation or locally calibrated Priestley–Taylor and Hargreaves–Samani equations; (c) accurate crop evapotranspiration (dual Kc approach); (d) the water (vertical and horizontal movements, effective rain) and nitrogen dynamics (soil organic matter mineralization) in the soil volume most effected by the root apparatus and water under drip irrigation regime taking into account row plant arrangement.

The DSS have specific features and checks for operating in NVZ (no leaching allowed, N application within maximum allowed for NVZ areas) and to adjust the growth curve in order to comply with limited N availabilities in NVZs.

With the aim of developing a tool easy to use by farmers, the DSS is based on a few easily accessible inputs both in the pre-season setup section and in the in-season management, also giving flexibility in the daily meteorological data requested, by selecting the method for evapotranspiration estimation which is compatible with the data available to the farmer. With the simplest option (Hargreaves) only minimum and maximum air temperature and rainfall are requested.

All these features, joined together in a DSS, represent a unique and useful tool for assisting vegetable growers in fertigation management.

## Description of the model

The software, designed to generate irrigation and N fertilization schedules in vegetable crops, combines several sub-models based on the daily calculation of crop dry weight accumulation, crop N uptake, N soil mineralization, crop evapotranspiration, and available soil, water, and N. It consists of three computational modules for (i) plant growth, (ii) water balance, and (iii) N balance.

### Plant growth

#### Shoot dry weight accumulation

To predict shoot dry weight (SDW) accumulation, *GesCoN* uses a non-linear logistic function based on thermal time as the independent variable:

(1) $$\text{SDW}=\beta_{1}/(1+e^{\left(\beta_{2}+\beta_{3}\;t\right)})$$

where β_1_, β_2_, and β_3_ are function parameters and *t* is the thermal time. Monteith (1977) introduced the concept of thermal time, calculated as growing degree days (GDD), which is the accumulation given by the summation of the daily averaged temperatures above a threshold called the base temperature.

(2) $$\text{GDD}=(T_{max}+T_{min})/2-T_{base}$$

where *T*_*max*_ and *T*_*min*_ are the daily maximum and minimum temperatures, respectively, and *T*_*base*_ is the minimum temperature threshold specific for a crop. In Equation (2) it is assumed that if on a given day [(*T*_*max*_ + *T*_*min*_)/2] > *T*_*base*_, then *GDD* > 0, but if [(*T*_*max*_ + *T*_*min*_)/2] < *T*_*base*_, there is no accumulation of thermal time and *GDD* = 0.

Biomass accumulation during the growing season for a vegetable crop harvested as a ripe fruit (e.g., peppers, pumpkin, squash, and type tomatoes) is generally well-represented by the logistic model (Weiner et al., 1998). The pattern follows a sigmoid curve that can be divided into three phases: (a) initial slow growth with an exponential trend; (b) rapid growth during midseason with a linear trend in which the highest SDW accumulation occurs, and (c) slow growth (approaching a plateau) late in the season. All these phases are present in a fruit vegetable crop, with phase (c) corresponding to fruit ripening. On the contrary, in a vegetable crop harvested as part of the plant in the vegetative stage (stems, leaves, roots) or even as a flower bud or immature fruit, the last part of curve (c) is not reached.

The parameter β_1_ represents the maximum asymptote of the logistic function, so if the product is harvested at the maturity stage, β_1_ can be calculated (in t ha^−1^) as the maximum SDW accumulation from the expected fresh yield by taking into account the harvest index (dry weight of harvested yield divided by the dry weight of the above ground biomass) and the dry matter content of the fresh yield at harvest, as follows:

(3) $$\beta_{1}=Exp\_yld\;YldDW/100/HI\;nplant$$

where *Exp_yld* is the maximum expected yield (g/plant), *YldDW* is the DW content in the fresh yield (g 100 g^−1^), *HI* is the crop harvest index and *nplant* is the number of plants per hectare.

#### Cycle specific adaptations of SDW modeling

#### Setting the initial growth starting point

*GesCoN* is intended to work with transplanted crops, vegetable crops established with transplants being those for which drip irrigation is most frequently used. The stage of plantlets at transplanting may have a strong effect on the following plant growth simulation, so it is important that a specific stage of plantlets is set.

When a crop is parameterized, the reference DW of plantlets (*Plts_Ref*) and the initial lag-phase growth rate (*Plts_GR*) must be defined. When a growing season is started with a crop, the initial value of plantlets DW (*Plts_DW*) must be given as input data in the plant growth setup module. If *Plts_DW* is lower or higher than the reference one (*Plts_Ref*), used in the calibration of that crop, the actual cycle of the crop is adjusted through a longer lag-phase or a shift forward of the cycle in order to align it with that used for calibrating the crop. However, plantlets must be in optimal condition (free from growth retardants) and must not exhibit signs of stress (i.e., elongated, etiolated, or bearing flowers).

#### Effects of thermal and water stresses on vegetative and reproductive growth

#### Thermal stress on SDW accumulation

As fertigation is normally used for warm season crops, the software assesses the negative effect on SDW accumulation of days with high stress temperatures. To account for the dependence of growth rate on high temperatures, *GesCoN* uses (a) a maximum temperature (*T_M1_* or temperature after which the crop starts suffering from high temperatures) and (b) a cut-off temperature (*T_M2_* or temperature above which the growth rate is zero; i.e., SDW accumulation is completely arrested by the stress conditions). Therefore, *GesCoN* allows deceleration of the growth rate when the temperature is beyond *T_M1_*, accounting for the negative effect of heat stress on plant growth. In *GesCoN* the relationship between these two point functions (*T_M1_* and *T_M2_*) is linear.

When maximum air temperature (*T*_*max*_) for a given day is between *T_M1_* and *T_M2_*, the GDDs calculated from Equation (2) are multiplied by a thermal stress coefficient (*K*_*ts*_) computed as follows:

(4) $$\begin{array}{l} K_{ts}=1-(T_{max}-T_{M1})/(T_{M2}-T_{M1})\\ \;\left[\text{if }T_{M1}<T_{max}<T_{M2}\right] \end{array}$$

It is assumed that if *T*_*max*_ > *T_M2_* then *K*_*ts*_ = 0 and if *T*_*max*_ < *T_M1_* then *K*_*ts*_ = 1, so it varies between 1 (no stress conditions) and 0 (maximum thermal stress). When *K*_*ts*_ < 1, it affects the growth of that day by proportionally reducing the accumulation of GDDs and consequently of SDW.

#### Thermal stress on plant fertility

If the crop under management is a fruit crop the temperatures during the flowering period can have an indirect effect on yield, through the number of fruits that are set on the plant.

During the flowering period, the software can estimate the adverse effect of high temperatures on fruit-set and pollination and thus on final yield and SDW accumulation. To perform this computation *GesCoN* requires the following parameters to be set: the beginning of flowering (in GDD) (*Flw_beg*), the duration of flowering (in GDD) (*Flw_dur*) and the maximum temperature level above which fruit setup is impaired (*Flw_Tmax*).

For a given day during the flowering period in which maximum air temperature is over the threshold level set for this period (*Flw_Tmax*), the software decreases the maximum attainable final dry weight (β_*1*_) as follows:

(5) $$\begin{array}{l} new\beta_{1}=\beta_{1}\;(1-(T_{max}-Flw\_Tmax)/Fl_{w\_dur})\\ \;[\text{if }T_{max}>Flw\_Tmax] \end{array}$$

where β_*1*_ and *new*β_*1*_ are the values of the foreseen final SDW accumulation (t ha^−1^), before and after the thermal stress on flowering. The subsequent SDW accumulation pattern is changed by affecting through the *new*β_*1*_parameter (which replaces the former β_*1*_) of the growth curve in Equation (1).

#### Water stress effect on SDW accumulation

Water stress can occur when (a) if for any reason a scheduled irrigation is postponed; (b) the wetting zone underneath the emitters created by an irrigation event is not ample enough to reach the root apparatus of neighboring plants. This latter condition may occur at the beginning of the crop cycle when plants are arranged in twin rows and the soil is too coarse. In this case root radial growth can still be limited and due to the limited retention capacity of the soil, the shape of the wet bulb could be too tapered, and not large enough to reach the plant roots.

Water stress occurs when on a given day the crop evapotranspiration is higher than the readily available water (RAW) (see below) on that day.

The crop response to water deficit in terms of reduction in SDW accumulation is estimated through a modification of the empirical approach proposed in the FAO Irrigation and Drainage Paper No. 33 (Doorenbos and Kassam, 1986). In the original formula the yield response to ET is expressed as:

(6) (1−Ya/Ym)=Ky (1−ETa/ETm)

where *Ym* and *Y* a are the maximum and actual yields, *ETm* and *ETa* are the maximum and actual evapotranspiration, and *Ky* is a yield response factor representing the effect of a reduction in evapotranspiration on yield losses.

In the modification we propose in *GesCoN*, the formula is applied to calculate the effect on SDW accumulation for a given day of water stress occurring on that day, as follows:

(7) $$\begin{array}{l} \left(1-SDW_{a}/SDW_{m})=Kts\;(1-ETc_{L}/(TAW-RAW)\right)\\ \;[\text{if }RAW_{a}<ETc] \end{array}$$

where *SDW*_*m*_ and *SDW_*a*_* are the maximum and actual SDW accumulation of that day, *ETc* is the estimated crop evapotranspiration, *RAW_*a*_* is the readily available water for that day and *Kts* indicates the average biomass response factor, which is the correlation factor between the reduction in evapotranspiration and the dry biomass loss. *ETc_*L*_* is the part of evapotraspiration occurring below the readily available water (RAW, see below) and TAW is the total available water (see below).

#### Calibration of the SDW accumulation pattern: the “SDWcheck” procedure

Measuring SDW accumulation during the crop cycle is recommended to improve the prediction accuracy of the growth model. The operation consists of performing an SDW measurement on a representative number of plants (depending on plant growth uniformity a random sampling from 10 to 20 plants per hectare is suggested) after about one-third of the growth cycle and inputting the data in a specific routine (“*SDWcheck*” procedure) of the DSS. On the basis of the difference between the observed and the predicted SDW accumulation, the software operates a fine tuning adjustment of the subsequent SDW accumulation pattern through two empirical relations for the calculation of:

(a) the new *T_M2_* threshold value:

(8) $$newT_{M2}=K_{T1}\;{\left(SDW_{o}/SDW_{p}\right)}^{2}+K_{T2}\;(SDW_{o}/SDW_{p})+K_{T3}$$

(b) the new expected maximum dry weight accumulation:

(9) $$new\beta_{1}=\beta_{1}\;[K_{SDW1}{\left(SDW_{o}/SDW_{p}\right)}^{KSDW2}]$$

where *SDW*_*o*_ and *SDW_*p*_* are the observed and the predicted aboveground SDW on the day of the *SDWcheck*, *K*_*T*1_, *K*_*T*2_, *K*_*T*3_, and *K*_*SDW*1_ and *K*_*SDW*2_ are function coefficients and β_*1*_ and *new*β_*1*_ are the values of the foreseen final SDW accumulation, before and after the *SDWcheck*, respectively.

A change in *T_M2_* has an effect for the subsequent days of the cycle on GDD computation and therefore on SDW accumulation, while a change in β_*1*_ (*new*β_*1*_ ≠ β_*1*_) has an effect on the subsequent SDW accumulation pattern by affecting the *new*β_*1*_ (which replaces the former β_*1*_ parameter) in the growth curve (Equation 1). The calibration of these two functions are assumed to be crop specific.

#### Root growth

The DSS only considers the most efficient part of the root apparatus for the computation of water and N balance. Thus, the effective root volume (ERV) is that portion of the root zone where the crop extracts the majority of water and nutrients under drip irrigation. In order to consider the ERV, the measurements of the maximum root deepening and enlargement are not set to the real physical growth values. In *GesCoN* it is assumed that the geometry of this part has a half-truncated ellipsoid form and its volume is computed as:

(10) $$ERV=\pi rr_{1}\;(2h/3)$$

where *r* is the maximum radial extension of the root apparatus and *r*_*1*_ is the radius orthogonal to *r*.

In order to simulate more pronounced root growth toward the wet soil beneath the neighboring emitters, the shape of the ERV is modified in a twin-row arrangement and in sandy soil. The modifications consist of the reduction of the radius *r*_*1*_, which is orthogonal to the axes connecting the center of the ERV and the neighboring emitter, and a shift of the center of the truncated ellipsoid (virtually the center of the ERV) toward the emitter point. The shift of the center is the same as the reduction in the root radius *r*_*1*_.

The root deepening and radial enlargement are simulated by the time-proportional linear increase from the initial to the maximum values. The initial and the maximum effective deepening (*Root_d_ini* and *Root_d_max*) and radial enlargement (*Root_r_ini* and *Root_r_max*) along with the time required to reach maximum values (*Root_d_max*) are input data which must be calibrated for each crop.

### Control of the cycle length

For a non-fruit crop the length of the cycle is defined by the achievement of a specific thermal sum (*TSMin*). In the case of a fruit crop the cycle is stopped if, for a given number of consecutive days (*d_SDWstop*) with positive GDDs, a very low increase in SDW occurs (lower than 0.4% compared to that of the previous day. This plateau phase corresponds to fruit ripening (crop maturity phase). However, the thermal sum should be between a minimum (*TSMin*) and a maximum (*TSMax*) value. This means that (i) a minimum value of thermal sum must be achieved by the crop before reaching the maturity phase, and that (ii) even if SDW accumulation is not on a plateau, the thermal sum must not exceed a maximum threshold. The values of *TSMin*, *TSmax*, and *d_SDW_stop* are crop-specific.

### The water balance

**Figure 1:**
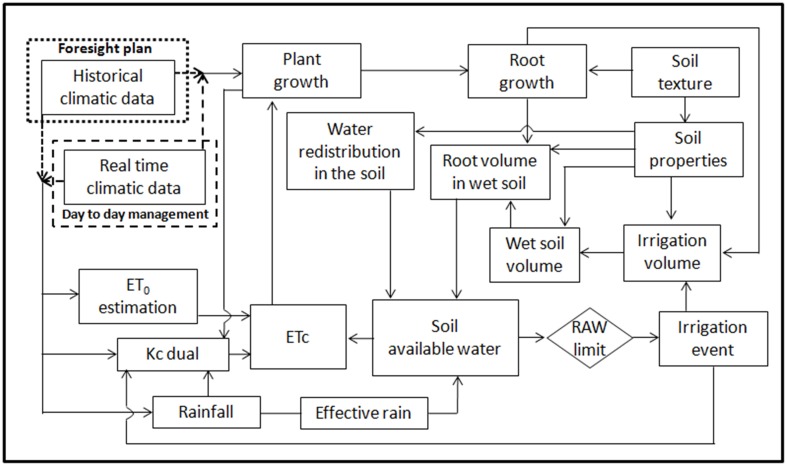
**Scheme of the main relationships considered in the water balance by** ***GesCoN***.

The DSS performs a daily water balance (Figure 1) keeping track of the soil water status by accounting for subtractions due to crop evapotranspiration and the main water additions (irrigations, effective rainfall, additional water in the supplemental soil volume reached by the growing roots and additional water from water movements due to its redistribution within the unsaturated soil) representing the total available water.

The Total Available Water (TAW) is computed as the amount of water in the root zone given by the difference between the water content at field capacity (θ_*FC*_) and wilting point (θ_*WP*_). The Readily Available Water (RAW) represents the fraction of TAW that a crop can extract from the root zone without suffering water stress (Allen et al., 1998).

When the water content in the wet root volume (WRV) (see below) is equal to or below the RAW fraction, irrigation is triggered and an amount of water is calculated in order to restore the water content to the field capacity.

#### Physical and hydraulic soil characteristics

The software requires some physical and hydraulic soil analytical data which are: soil texture, bulk density, field capacity (θ_*FC*_), permanent wilting point (θ_*WP*_), and saturated hydraulic conductivity (*Ks*). If no specific analytical data are available for θ_*FC*_, θ_*WP*_, or bulk density, *GesCoN* also offers the possibility of estimating these soil parameters from soil texture using one of the nine pedo-transfer functions included in a specific routine of the software. Among these functions, the DSS has the values proposed by Saxton et al. (1986) as default settings (Table 1).

**Table 1:** **Mathematical expression for the selected pedotransfer functions used by** ***GesCoN***.

**Method#**	**Mathematical expressions**	**Authors**
1	θ_*FC*_ = 0.3486–1.8 × 10^−3^ *Sa* + 3.9 × 10^−3^ *Cl* + 0.0228 *OM*–0.0738 *BD*	Rawls et al., 1983
	θ_*WP*_ = 0.0854–4 × 10^−4^ *Sa* + 4.4 × 10^−3^ *Cl* + 0.0122 *OM*–0.0182 *BD*	
2	θ_*FC*_ = exp(-3.43 + 0.419 (*Cl* + *Si*)^0.5^–1.83 × 10^−3^ (*Cl* + *Si*)^1.5^)	Hutson. (reproduced from Hutson and Wagenet, 1992)
	θ_*WP*_ = exp(−4.384 + 0.404 (*Cl* + *Si*)^0.5^–9.85 × 10^−7^ (*Cl* + *Si*)^3^)	
3	θ_*FC*_ = 0.73426–1.45 × 10^−3^ *Sa*–0.29176 *BD* if *Sa* = 75	Manrique et al., 1991
	θ_*FC*_ = 0.5784 + 2.27 × 10^−3^ *Cl*–0.28438 *BD* if *Sa* < 75	
	θ_*WP*_ = 0.02413 + 3.73 × 10^−3^ *Cl*	
4	θ_*FC*_ = 0.2668 + 3.9 × 10^−3^ *Cl* + 1.3 × 10^−3^ *Si* + 4.6 × 10^−3^ *OM*–0.0764 *BD*	British soil service. (reproduced from Hutson and Wagenet, 1992)
	θ_*WP*_ = 0.0611 + 4 × 10^−3^ *Cl* + 5 × 10^−4^ *Si* + 5 × 10^−3^ *OM*	
5	θ_*FC*_ = 10^−2^ × (11.83 + 0.96 *Cl*–0.008 *Cl*^2^)	Petersen et al., 1968
	θ_*WP*_ = 10^−2^ × (1.74 + 0.76 *Cl*–0.005 *Cl*^2^)	
6	θ_*FC*_ = (0.043 + 0.004 *Cl*)/(0.471 + 0.00411 *Cl*)	Bruand et al., 1994
	θ_*WP*_ = (0.008 + 3.67 × 10^−3^ *Cl*)/(0.471 + 0.00411 *Cl*)	
7	θ_*FC*_ = 10^−2^ × *BD* (2.65 + 1.105 *Cl*–0.01896 *Cl*^2^ + 1.678 × 10^−4^ *Cl*^3^ + 15.12 *BD*–6.745 *BD*^2^–0.1975 *Cl* × *BD*)	Canarache, 1993
	θ_*WP*_ = 10^−2^ × *BD* (0.2805 *Cl* + 9.615 × 10^−4^ *Cl*^2^)	
8	θ_*FC*_ = 10^−2^ × (20.81 + 0.45 *Cl* + 0.13 *Si*–5.95 *BD*)	Hall et al., 1977
	θ_*WP*_ = 10^−2^ × (1.48 + 0.84 *Cl*–0.0055 *Cl*^2^)	
9^*^	*a* = exp(−4.396 − 0.0715 *Cl*–4.88 × 10^−4^ *Sa*^2^ − 4.285 × 10^−5^ *Sa*^2^ × *Cl*)	Saxton et al., 1986
	*b* = (−3.14 − 2.22 × 10^−3^ *Cl*^2^–3.484 × 10^−5^ *Sa*^2^ × *Cl*)	
	*sat* = 0.332–7.251 × 10^−4^ *Sa* + 0.1276 Log(*Cl*)	
	θ_*WP*_ = (15/a)^(1/*b*)^	
	θ_*FC*_ = (0.333/a)^(1/*b*)^	
	*BD* = (1 − sat) 2.65	

θ_*WP*_, soil water content at permanent wilting point; θ_*FC*_, soil water content at permanent field capacity; Cl, % of clay content; Si, % of silt content; Sa, % of sand content; BD, bulk density (g cm^−*3*^); OM, % organic matter content; Sat, saturated hydraulic conductivity.

* Default setting by the software.

If *Ks* is not available as analytical data, a default value is assigned by the software, using the values proposed by Carsel and Parrish (1988) for the 12 USDA soil textural classes.

Alternatively, all these soil characteristics can be estimated by using more specific and comprehensive software such as *SOIL*PAR (Acutis and Donatelli, 2003) or ROSETTA (Schaap et al., 2001).

#### Estimation of water losses

#### Reference evapotranspiration

To define the crop water consumption, *GesCoN* uses reference crop evapotranspiration (ET_0_), proposing several alternatives for its estimation. When available from a nearby weather station, the daily ET_0_-value can be an input value. If it is not available it can be computed by the DSS from weather data using one of three different methods: (i) the Penman–Monteith model (PM) from Paper No. 56 of FAO (Allen et al., 1998), (ii) the Priestley–Taylor model (PT) (Priestley and Taylor, 1972), and (iii) the Hargreaves–Samani model (HS) (Hargreaves and Samani, 1985). The purpose of the different alternatives is to give flexibility in selecting the method which is compatible with the available meteorological data. The PM method has a strong theoretical basis, including energy balances to model ET_0_, but requires many daily input data: maximum and minimum temperature, solar radiation, maximum, and minimum relative humidity (or dew-point temperature), and wind speed. It is considered the best method for estimating daily ET_0_ in all climates and this assumption has been confirmed by a lot of research in the last decade (Hargreaves and Allen, 2003; Tyagi et al., 2003; Delghani Sanij et al., 2004; Berengena and Gavilan, 2005; Trajkovic, 2005, 2007; Gavilan et al., 2006; Lopez-Urrea et al., 2006; Trajkovic and Kolakovic, 2009).

As stations with detailed meteorological parameters are not always readily available, the software provides the option of using the PT or HS methods as an alternative.

In the PT method the aerodynamic term of the PM equation is replaced by a dimensionless empirical multiplier (α coefficient), so the method can be used when data for the aerodynamic term (relative humidity, wind speed) are unavailable. The PT equation can be written as:

(11) $$ET_{\text{0},\text{PT}}=\alpha \;(s/(s+\gamma ))(Rn-G)1/\lambda$$

where ET_0, PT_ is the estimated reference evapotranspiration from the PT equation (mm day^−1^), λ is the latent heat of vaporization (MJ kg^−1^), *s* is the slope of the saturation vapor density curve (kPa °C^−1^), γ is the psychrometric constant (kPa °C^−1^), *Rn* is net radiation (MJ m^−2^ day^−1^), *G* is soil heat flux (MJ m^−2^ day^−1^), and α is a model coefficient. The α coefficient depends on the advectivity of the environment and can vary from 0.6 for wetlands to 2.47 for arid or semi-arid climates (Cristea et al., 2013) and it may also have a seasonal variation (de Bruin and Keijman, 1979). The calibration of the α coefficient for the specific area and for the specific time period of the year is therefore advisable in order to improve ET_0, PT_ estimates. The PT equation also needs the input of the soil-plant reflection coefficient for solar radiation (*albedo*). The *albedo* coefficient varies with the soil type, its humidity, with the type of vegetation, with the sun angle during the day. For simplicity in this version of *GesCoN* a value of 0.24 is the default setting in the software, according to the average values indicated for a field crop in Evett (2002). The *albedo* value can be however changed in the crop setup module.

The HS model is proposed as a less input-requiring approach. It only requires minimum and maximum air temperatures, thus representing the simplest approach for estimating ET_0_ on a commercial farm.

The HS equation can be written as:

(12) $$ET_{0,HS}=C_{H}\;{\left(T_{\text{max}}-T_{\text{min}}\right)}^{EH}(T_{\text{mean}}+C_{T})\;Ra$$

where ET_0, HS_ is the estimated reference evapotranspiration from the HS equation (mm day^−1^); *Ra* is the calculated extraterrestrial radiation (MJ m^−2^ day^−1^); *T*_max_, *T*_min_, and *T*_mean_ are the daily maximum, minimum and mean air temperature (°C), with *T*_mean_ calculated as the average of *T*_max_ and *T*_min_; *C_*H*_*, *E_*H*_*, and *C_*T*_* are empirical coefficients, which in the original Hargreaves–Samani formulation are: *C_*H*_* = 0.0023, *E_*H*_* = 0.5, and *C_*T*_* = 17.8 (Hargreaves and Samani, 1985).

Although the HS model has the advantage of requiring few daily meteorological data (only air temperatures), this model is reportedly indicated to overestimate ET_0_ especially at humid locations and that the adjustment of the equation coefficients to local conditions is necessary to improve its estimation (Jensen et al., 1990; Amatya et al., 1995; Itenfisu et al., 2003; Temesgen et al., 2005; Trajkovic, 2005). It is thus most appropriately applied to climates similar to that where it was developed.

In order to cope with the above limitations in the ET_0_ estimation accuracy in HS an PT equations, both requiring site specific calibration, *GesCoN* has a specific routine that allows the calibration of the equations to the local climatic conditions. Calibration can be performed using the PM estimates as reference values. PM is, indeed, recommended by the FAO as a reference to verify other empirical methods (Allen et al., 1998). Historical climatic data from a meteorological station near to the site are needed to perform the calibration to use PM. The specific window period of the year relative to the crop cycle can be used for the calibration and it is possible to select from 1 to 5 years of datasets of meteorological data to calibrate the equations.

**Figure 2:**
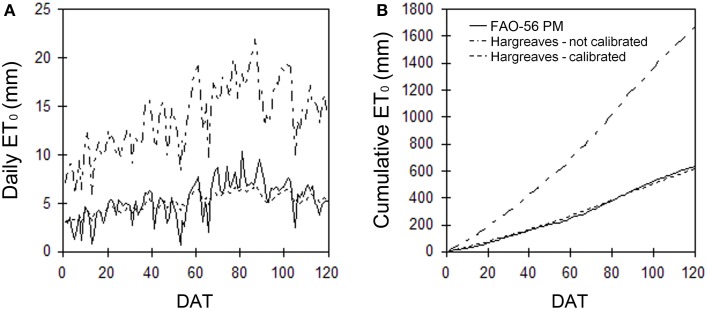
**Example of Hargreaves over Penman-Monteith estimates as daily ET_0_-value or season cumulative ET_0_ before and after calibration**.

The routine involves the adjustment of the *E_*H*_* parameter of the HS model, and of the α coefficient of the PT model with a fitting procedure between the cumulative ET_0_ patterns estimated by the PM and those estimated by the HS or the PT models. The convergence of the curves is obtained by finding the *E_*H*_* and the α-value for each year that minimizes the sum of squares of the differences from the PM estimates. The goodness of fit of the cumulative evapotranspiration during the seasons obtained from the HS or PT calibrated models over the PM estimates is evaluated graphically and by the percentage Root Mean Square Error (%RMSE). The averaged coefficient values over the years, resulting from the calibrations, are considered by the software as new calibrated parameters. The routine also gives a graphical output of the PM and HS or PT estimates, displaying the adaptation of the non-calibrated and calibrated model patterns over the PM estimates (Figure 2).

**Figure 3:**
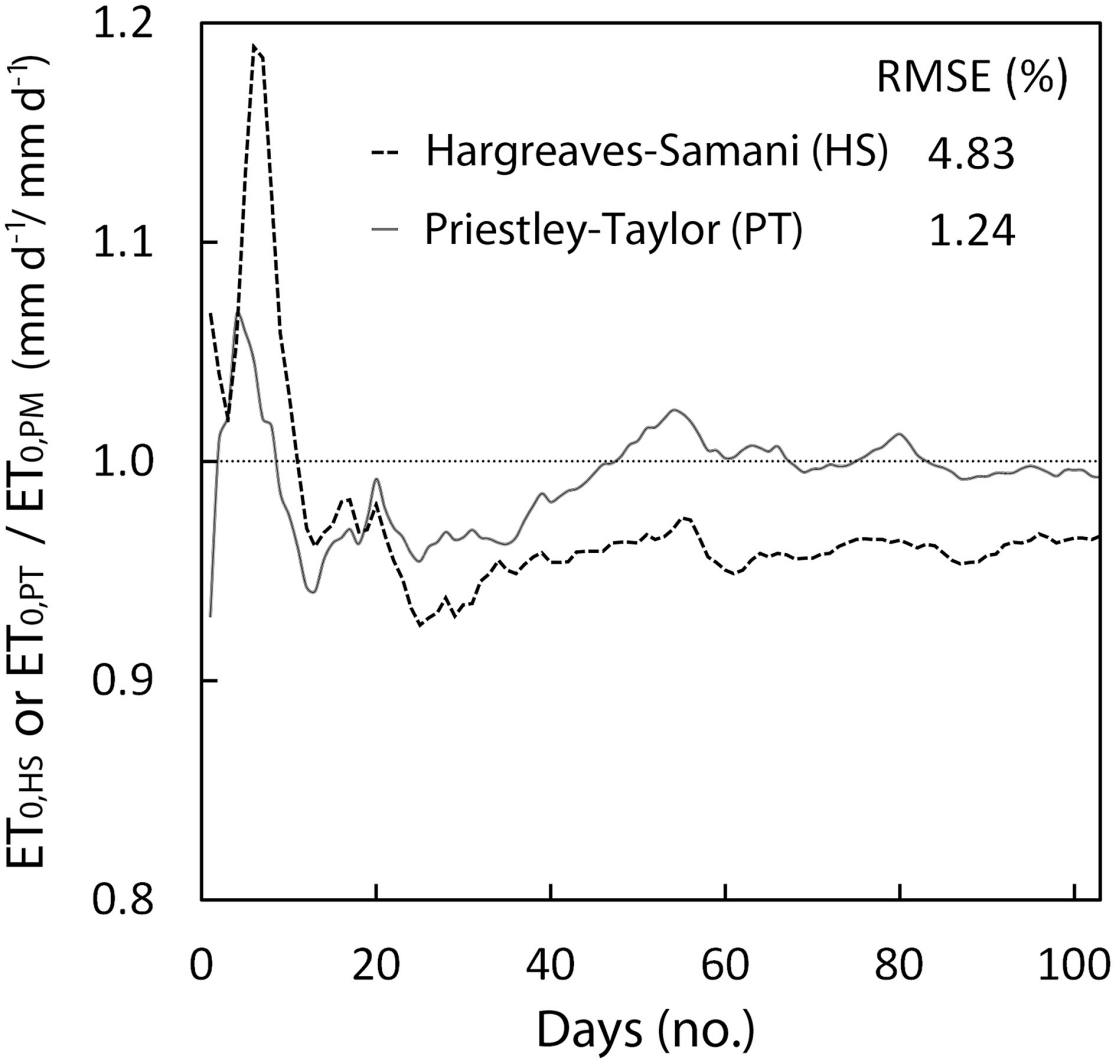
**Deviations in daily ET_0_ estimates of the Hargreaves and Priestley-Taylor model, after being calibrated for the area, compared with the Penman–Monteith model**. The test relates to a typical period for a processing tomato crop in the Foggia area (Southern Italy) and is given as an example of the accuracy of the Hargreaves and Priestley-Taylor model after site calibration.

The three approaches (PM, PT, and HS) are widely used for practical applications and have been validated and compared in a number of scientific papers (Yoder et al., 2005; Sentelhas et al., 2010; Fisher and Pringle, 2013). However, a comparison between ET_0_ estimated through PM and the other two methods has been analyzed as an example of the effect of the calibration procedure of *GesCoN*. After calibrating the PT and HS equations with a dataset of five previous years of meteorological data for the same site, the equations were compared with the PM function in a different year. The comparison concerned a specific period (from 10/Jun/2005 to 20/Sep/2005) on the Capitanata plain (Foggia, Italy). Figure 3 shows the ratio between the estimates of ET_0_ obtained through HS (ET_0, HS_) or PT (ET_0, PT_) and the PM (ET_0, PM_) equation. It can be observed that even if PT gave a better performance compared with HS, in both cases the estimation was more than acceptable, the %RMSE of deviations from PM being lower than 5%.

#### Crop evapotranspiration

The software can selectively use the single or the dual crop coefficient (*Kc*) approach for estimating crop evapotranspiration (ETc) from reference evapotranspiration.

In the single crop coefficient approach, the effect of crop transpiration and soil evaporation are combined into a single Kc coefficient. While the “dual” Kc methodology consists of a separate computation of the two components of ETc: (a) the plant transpiration from crops having a dry soil surface, calculated using the basal crop coefficient (*Kcb*, which depends on crop species and its development stage), and (b) the soil evaporation from bare soil based on the water balance of the soil surface layer, calculated using the soil evaporation coefficient (*Ke*) (Allen et al., 1998). Therefore, potential (maximum) ETc can be derived from reference evapotranspiration (ET_0_) as:

(13) $$ETc=(Kcb+Ke)\;ET_{0}$$

Both the *Kc*- and *Kcb*-values are reported in the FAO Paper No. 56 corresponding to the three growth stages for many vegetable crops (initial, middle and late, hence *Kc_ini*, *Kc_mid*, *Kc_end*), which reflect the changing rates of crop-water use over the growing season. Different environmental conditions between regions can cause variation in crop growth, which affects the *Kc* and *Kcb*-values (Allen et al., 1998).

In order to compute the ETc, *GesCoN* calculates transpiration by appling *Kcb* on the part of the soil surface covered by vegetation which is assumed to be coincident with the top area of ERV. Evaporation is calculated by applying *Ke* only on the soil surface of the wet bulbs (WB – see below) created after an irrigation. Evaporation is calculated on the wet surfaces.

When the crop is grown on plastic mulch, *GesCoN* can consider the effect of mulch associated with the reduction in evaporation from the soil surface due to the presence of the plastic film and with the increase in transpiration from vegetation caused by the transfer of both sensible and radiative heat from the surface of the plastic cover and by the higher vegetative growth of mulched plants (Allen et al., 1998). To take into account these effects *GesCoN* allows modification of the soil evaporation (*Ke*) and transpiration (*Kcb*) components used in the dual *Kc* approach, by reducing the first and increasing the second. The percent of *Ke* reduction (*Mulch_Ke*) and of *Kcb* increase (*Mulch_Kcb*) needs to be calibrated on a crop. Values of 65% for *Mulch_Ke* and of 20% for *Mulch_Kcb*, reported by Allen et al. (1998) as the average of various horticultural crops, are the default settings in the DSS.

#### Estimation of water additions

#### Computation of wet soil volume and of available water

Soil humidity is not homogeneously distributed under drip irrigation. The shape and volume of the wetted soil and the spatial distribution of soil water under drip irrigation vary with soil hydraulic properties, emitter discharge rates, spacing, irrigation quantity and frequency, crop water uptake rates and root distribution patterns (Subbaiah, 2013).

The software assumes that humidity resulting from irrigation water is concentrated in the wet soil volumes (wet bulbs – WB) under the emitters along the irrigation pipes. To calculate the volume of the WB, the software assumes that their geometry is represented by a truncated ellipsoid as suggested by Zur (1996). The semi-empirical approach suggested by Schwartzman and Zur (1986), and validated by Ainechee et al. (2009), is used to assess the width and the depth of the wet soil volume. The approach relates the measurements of a wet bulb to the emitter discharge, the saturated hydraulic conductivity (*Ks*) of the soil and the volume of water in the wet soil volume as follows:

(14) $$Xf=1.82\;{\left(Vw\right)}^{0.22}{\left(Ks/q\right)}^{-0.17}$$

(15) $$Zf=2.54\;{\;(Vw)}^{0.63\;}{\left(Ks/q\right)}^{0.45\;}$$

where *Xf* and *Zf* are, respectively, the diameter and the depth (m) of wet soil volume beneath each emitter, *Vw* is the volume of applied water (m^3^ ha^−1^), *Ks* is the saturated hydraulic conductivity (m s^−1^), and *q* is the emitter flow rate (m^3^ s^−1^).

In our further simplification, it is assumed that just after irrigation all the wetted soil volume under the emitter point is at field capacity so the dimension of the truncated ellipsoid is recalculated in order to match this requirement as follows:

(16) $$Xf_{1}={\left((3\;Vw\;Xf)/(2\;\pi \;FC\;Zf)\right)}^{1/3}$$

(17) $$Zf_{1}=Xf_{1}\;Zf/Xf$$

*GesCoN* calculates the TAW in the ERV (see below) accounting for the following components:

(18) TAW=Qirr+Qrain+Qred+Qadd

where *Qirr* is the amount of water after irrigation, *Qrain* is the effective rain (the part of rainfall that is stored in the root volume and not lost by surface runoff or deep depletion), *Qred* is the water available thanks to redistribution movements (see below) and *Qadd* is the water reached by the new growth of the root system following its deepening and enlargement into the soil.

#### Available water from irrigation

**Figure 4:**
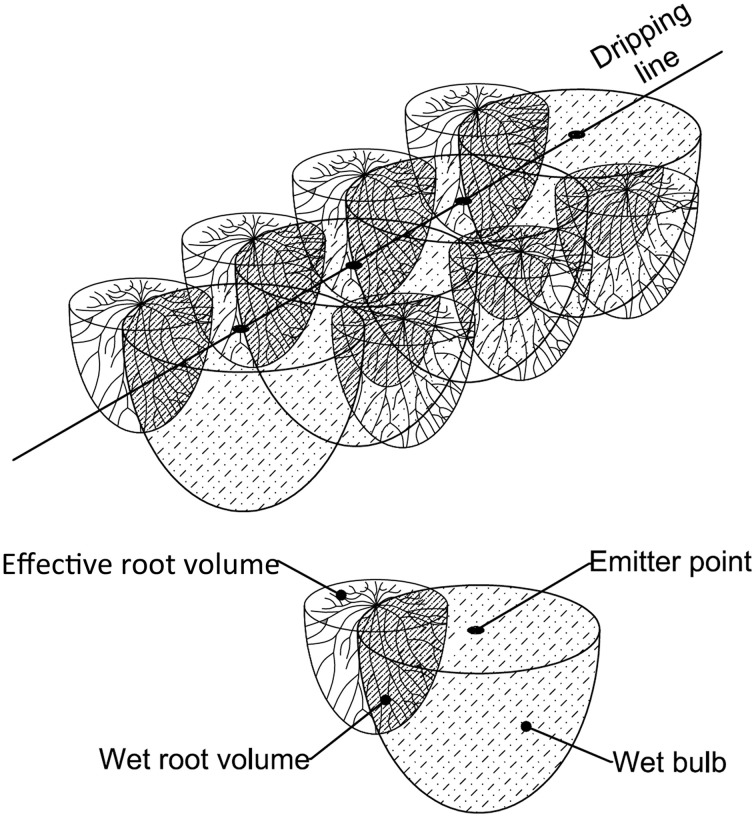
**A three-dimensional representation of the physical model used by GesCoN to simulate the interaction between root apparatus and the wet soil volume under an emitter source point**. The case refers to the interaction of a drip line with two twin rows.

To calculate *Qirr* in ERV, the software computes the part of ERV for each single plant which interacts with the neighboring wet bulbs (Wet Root Volume – WRV) (Figure 4), this is calculated by considering the sum of the soil volumes of the interaction between ERV and WB.

On a given day of the cycle, the number of WBs and their relative capacity to supply water to a plant depends on ERV and WB dimensions (radius and depth). ERV depends on the plant growth phase and on soil texture, while WB dimensions are related to (i) emitter and plant spacing (ii) plant arrangement (single row or twin rows), and (iii) soil texture.

Having computed the average plant WRV, *GesCoN* then estimates the total available water to a plant from an irrigation as:

(19) $$Qirr=(\theta_{FC}-\theta_{WP})\;WRV$$

where θ_*FC*_ is the water content at field capacity and θ_*WP*_ the water content at wilting point.

#### Estimation of effective rainfall

In the case of rain during the cycle, and if no plastic mulch is used, the rainfall value must be recorded in the proper daily data input section. Considering the high spatial variability of rain it is preferable to consider the amount of water (mm) collected in a rain gauge installed on the field. Alternatively the rainfall recorded at a nearby weather station can be used.

To compute the effective rainfall the DSS considers the part of rainfall that is stored in the ERV and not lost by surface runoff or deep depletion:

(20) Qrain=Rainfall−runoff−depletion.

The Soil Conservation Service (SCS) Runoff Curve Number (CN) method, developed by the USDA Natural Resources Conservation Service (USDA-SCS, 1985), is used to compute the amount of water lost by runoff. It is based on the hydrological soil group, land use, treatment, and hydrological condition of the area and is expressed as:

(21) Q=(P−0.2S)2/(P+0.8S)  [when P>0.2S]

(22) Q=0  [when P≤0.2S]

(23) S=(1000/CN−10)25.4

where *Q* is the direct runoff or rainfall excess (mm), *P* is the rainfall (mm), *S* is the maximum potential soil water retention (mm) and *CN* is curve number parameter (dimensionless).

The vertical drainage of rain water following a storm is computed as the fraction of the rain water content above field capacity calculated both on the ERV and WB.

#### Water redistribution in unsaturated soil conditions

The redistribution of water inside the WB toward WRV in the days following an irrigation event is considered by the DSS accounting for water movement in the horizontal direction. This movement is due to the different soil pressure head between these two adjacent zones, created by the water subtracted from the roots and evaporated in the WRV. The approach proposed in the model is an approximate method to evaluate water transfer in unsaturated soil conditions in the crop-soil system.

The model adopts a simple procedure using an integration strategy over the soil zones in a daily time step, considering the soil hydraulic functions calculated according to van Genuchten (1980) and Mualem (1976):

(24) $$S=\frac{\theta -\theta_{r}}{\theta_{s}-\theta_{r}}={\left[\frac{1}{1+{\left|\alpha h\right|}^{n}}\right]}^{m}$$

(25) $$K(\theta )=Ks\;\sqrt{\text{S}}\;{\left[\text{1-}({\text{1-S}}^{\frac{1}{\text{m}}})\right]}^{2}$$

where *S* is the relative saturation, θ*s* and θ*r* are the saturated and residual soil water contents, α (cm^−1^) and *n* are the shape parameters of the retention and conductivity functions, *m* = 1 – 1/*n*, *Ks* (cm d^−1^) is the saturated hydraulic conductivity and *h* is the pressure head.

On a daily time step the soil water flow (*q*) between two adjacent zones is approximated as:

(26) $$q=K(\theta )\left(S_{1}-S_{2}\right)(\theta_{s}-\theta_{r})Ve$$

where *S*_1_ and *S*_2_ are the relative saturation of the adjacent zones, *K(*θ*)* is calculated considering the average soil water content (*θ)* of the two adjacent zones and *Ve* is the wetted volume in the effective root zone.

#### Irrigation management

As a general rule, if irrigation is performed in a Nitrate Vulnerable Zone (NVZ), the software does not allow a wet bulb depth greater than the maximum rooting depth, in order to prevent water depletion.

On the first day of the cycle, irrigation is started by default. During the cycle, an irrigation event is triggered when the loss of water by evapotranspiration in the WRV exceeds the threshold value of the RAW. RAW depends on the crop and in vegetables can vary from 20 to 50% of TAW (Allen et al., 1998). The amount of irrigation water is computed in order to restore the water content in the WRV to field capacity. However, *GesCoN* calculates the amount of irrigation water with different decision rules depending on the growing phase of the crop:

- *Stand establishment – initial root growth:* this period is between the transplanting day and the following high root deepening phase. The software defines the water volume on the basis of the radius of wet bulb which must at least reach the center of the root zone of all of the neighboring plants. The radius depends on (i) soil texture, (ii) the distance between emitters and plants, and (iii) the plant arrangement used (single or twin rows). Water irrigation volume may be critical in this phase in a twin rows arrangement and in sandy soil conditions. It could be too low if no water depletion is imposed (e.g., NVZ management). In this case wet bulb radius could not be long enough to reach the neighboring plantlets' ERV. On the contrary if no limit on WB depth is imposed (e.g., non-NVZ management), this could exceed the maximum rooting depth causing large water depletion.
- *Root deepening:* in this period fast root deepening occurs until the maximum depth is reached and the software defines the water volume on the basis of WB depth which must be equal to root depth.
- *Full root development:* this period starts when the root apparatus reaches the maximum depth, and, if the crop is a fruit crop, ends when crops reach maturity. In this period the software sets the irrigation volume in order to have a WB depth equal to maximum rooting depth.
- *Fruit ripening phase:* this phase is only present in a fruit crop, it is absent if the crop is harvested at a vegetative growth or immature fruit stage. During this period irrigation is stopped.

An extra water volume can be considered by the DSS to account for uneven distribution by the irrigation system. The amount of additional water is calculated by increasing the irrigation water volume by a given percentage. The percentage of water increment is an input value (*IrrPlus*, defaut value = 0) estimated by the user and depends on the uniformity of water distribution by the drip irrigation system used.

### The N balance

**Figure 5:**
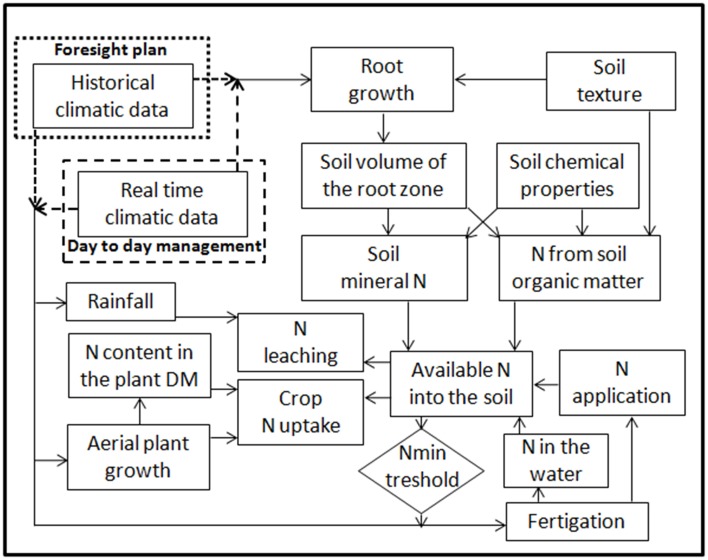
**Scheme of the main relationships considered for the N balance by** ***GesCoN***.

The methodological approach is based on a daily N balance between N uptake by the crop and N available in the root zone, the difference is the amount of N fertilizer that should be applied to the soil in order to fulfill N-crop requirements (Figure 5). The N balance is computed every day, considering crop N uptake as subtraction from the soil root zone or depletion due to rain, and the following as additions: (i) the mineralization of SOM., (ii) the increase of interception of the soil mineral N stock present at the beginning of crop cycle due to root growth, (iii) the application of any nitrates present in the irrigation water, (iv) the application of inorganic N-fertilizer. For the sake of simplicity, in order to limit the input of environmental parameters, and also considering the brevity of the growing season in a vegetable crop, the program also assumes that deposition of N from the atmosphere (non-symbiotic fixation, wet and dry deposition) is equal to gaseous emissions (volatilization, denitrification) and thus these two terms are not considered. N leaching following heavy rain or excessive irrigation is calculated, while no N leaching occurs under NVZ management irrigation regimes, with depletion being prevented by the precise control of the depth of the wetting front.

The program assumes that all N-fertilizer supplied by fertigation is readily available to the crop.

The following chemical analytical data are needed at the beginning of the crop cycle: the soil concentration of nitrate-N and ammonium-N, to calculate the mineral soil N available at the beginning of the crop, and the soil concentration of organic matter (SOM), total N, total calcium carbonate, to calculate the N mineralization from SOM during the crop cycle. The nitrate-N concentration in irrigation water is also required to account for N addition through irrigation.

#### N crop uptake

The N uptake by the crop can be estimated by considering the N concentration in the dry mass at each point in the growth cycle. Lemaire and Salette (1984) developed the concept of critical N concentration in shoot biomass. This value corresponds, at any moment of vegetative growth, to the minimum concentration of N necessary to achieve the maximum aboveground biomass. This concentration is represented by the power equation:

(27) $$\%N=a\;SDW^{-b}$$

where *SDW* is the total shoot dry biomass expressed in t·ha^−1^, *%N* the total N concentration in shoot biomass expressed as a percentage of the shoot dry matter, *a* represents the N concentration in the dry biomass when DW = 1 Mg ha^−1^, and *b* is a statistical parameter governing the slope of the relationship. The coefficients *a* and *b* are considered crop specific and many studies have been carried out to determine these parameters for various crops (Jeuffroy et al., 2002). The relationship between crop N-uptake and accumulated dry matter on the shoot biomass is described by the allometric relation derived from Equation (28):

(28) $$Nupt=10aSDW^{1-b}$$

#### Computation of the available soil N

##### N from soil organic matter mineralization

Organic matter dynamics is modeled according to the model of Hénin and Dupuis (1945), modified and validated by Boiffin et al. (1986) and Mary and Guérif (1994).

The mineralization coefficient (*k*_2_) is estimated daily on the basis of soil texture, limestone content and air temperature using Equation (29)

(29) $$k_{2}=1200/(clay+200)(0.3\;CaCO_{3}+200)(T_{Mean}/2-5)$$

where *clay*, *CaCO_3_* and *T_*Mean*_* represent soil clay and calcium carbonate content (g·kg^−1^) and mean daily air temperature (°C) from the local meteorological station, respectively.

In order to compute the daily N-mineral (*NSOM*) delivered by the soil organic matter mineralization the following equation is used:

(30) $$NSOM=(Soil_{wt}\;Norg/1000\;k_{2}\;P)/365$$

where *Norg* is the organic N concentration (g kg^−1^), *k_2_* is the mineralization coefficient, *Soil_*wt*_* is the soil mass containing most of the root apparatus (kg ha^−1^), *P* is a modifier of the mineralization coefficient (dimensionless) calculated as:

(31) $$P=f_{r}\;I\;Ts$$

where *f_*r*_* is a coefficient considering crop management (Table 2), *I* is a mineralization weight factor (suggested value: 1.25), *Ts* is a tillage factor (1.0 if the soil is tilled at least once every 4 years; 0.5 if no tillage practices have been used in the last 4 years; 0.8 in intermediate cases, with at least 1 year of minimum tillage).

**Table 2:** **Crop management coefficient (*****fr*****) used to consider crop management in the calculation of the modifier of the mineralization coefficient (*****P*****) in the organic matter indicator (from Bockstaller and Girardin, 2000)**.

**Crop residue management**	**Organic input frequency (manure, compost, etc.)**			
	**>10 years**	**Between 5 and 10 years**	**Between 3 and 5 years**	**<3 years**
Removed or burned	0.8	0.9	1.0	1.1
Incorporated once every 2 years	0.9	1.0	1.1	1.2
Incorporated every year	1.0	1.1	1.2	1.3

#### Soil N mineral stock interception

An increase in the interception of the mineral N already present at the beginning of the crop occurs on each day due to root growth. It is computed daily by multiplying the nitrate-N and ammonium-N contents which result from soil analysis at the beginning of the crop cycle with the increase in the soil volume explored by the roots in that day. When root growth reaches its maximum values, no further increase in soil N mineral stock interception is computed by the software.

### N fertilization management

If the N availability in the root volume drops below the minimum N threshold the software triggers an application of N fertilizer. The threshold is an input value which is crop-specific and depends on the crop stage – three levels for this threshold are foreseen: ini-, mid- and final-season (*N_min_res_1*, *N_min_res_2* and *N_min_res_3*, respectively). The amount of N application is calculated on the basis of the N crop uptake until the next presumable irrigation, minus the simultaneous release of N from SOM mineralization or interception of the N mineral stock. This amount is increased or decreased with a *K_*Nrate*_* coefficient depending on the stage of the crop. Indicating the days from transplant (DAT) with *T*_1_ for the end of the initial phase, with *T*_2_ for the beginning of mid phase, with *T*_3_ for the end of the mid phase and with *T*_4_ the presumable end of the cycle, *K_*Nrate*_* is 1.2 in the initial phase (DAT = *T*_1_), 2.5 in the phase of linear and rapid growth (*T*_1_< DAT = *T*_2_), 0.7 in the first 60% of the mid phase, 0.3 in the last 40% of the mid phase, and 0 after the mid phase (DAT > *T*_3_).

## How the DSS works

The *GesCoN* interface has a Windows screen format with a menu bar provided at the top of the screen to access input and other functional screens. The *GesCoN* interface can be divided into two main components: sector setup, and fertigation manager with several utility routines.

### Sector setup

The fertigation sector setup is an input section created by the software for different crops, cultivars or crop cycles, and different types of soil on a farm. So the *GesCoN* can create and manage different fertigation sectors within a farm. The sector setup is the user interface for setting and modifying the initial *GesCoN* parameters classified in 13 input pages (Table 3). The entries on the sector setup are only required at the beginning of the crop season.

**Table 3:** **Entries and parameters used by** ***GesCoN*** **to work on a crop**.

**Setup page**	**Entry/parameter**		**Meaning**	**Type^a^**
	**#**	**Name**		
Sector info	1	*Sec_ID*	Sector I	SS
	2	*Lat*	Latitude	SS
	3	*Elev*	Elevation (m)	SS
	4	*Meteofile*	File name with the historical meteorological data. (this file must be previously prepared by the user in order to allow the DSS to operate in simulation mode).	(SS)
	5	*NVZ*	NVZ area (Y/N)	SS
Soil chemical profile	6	*Soil_SO*	Soil organic matter content (mg kg^−1^)	SS
	7	*Soil_Norg*	Soil organic N content (mg kg^−1^)	SS
	8	*Soil_NO3*	Soil NO_3_-N content (mg kg^−1^)	SS
	9	*Soil_NH4*	Soil NH_4_-N content (mg kg^−1^)	SS
	10	*CaCO3*	Soil total CaCO_3_ content (mg kg^−1^)	SS
Soil physical properties	11	*Cl*	Soil clay content (g kg^−1^)	SS
	12	*Si*	Soil silt content (g kg^−1^)	SS
	13	*Sa*	Soil sand content (g kg^−1^)	SS
	14	*SoilClass*	USDA soil classification	AS
Crop profile	15	*Species*	Species common nam	US
	16	*Cv*	Cultivar name	US
	17	*In_row*	Plant to plant spacing (cm)	US
	18	*Bet_rows*	Between rows distance (cm)	US
	19	*Bet_twins*	Between twins distance (cm)	US
	20	*Planting_d*	Planting date (date)	US
	21	*Exp_yld*	Expected fresh yield (g/plant)	C
	22	*Pl_arr*	Plant arrangement (single or twin row)	US
	23	*Mulch*	Presence of film mulching (Y/N)	US
	24	*Mulch_Ke*	*Ke* reduction with film mulching (%)	C
	25	*Mulch_Kcb*	*Kcb* increase with film mulching (%)	C
	26	*YldSDW*	Dry mass content in the fresh yield at harvest (%)	C – L
	27	*HI*	Harvest index	C – L
	28	*d_SDWstop*	Days with low SDW increment before full maturity (no.)	C
	29	*TSMin*	Minimum thermal sum for crop maturity (°Cd)	C
	30	*TSMax*	Maximum thermal sum for crop maturity (°Cd)	C
	31	*RAW*	Readily available water (% of *TAW*)	L
Irrigation setup	32	*Lines_Dist*	Drip lines spacing (cm)	US
	33	*Emit_Dist*	Emitter spacing (cm)	US
	34	*E_Disch*	Emitter discharge rate (L/h)	US
	35	*IrrEff*	Irrigation system efficiency (%)	US
	36	*MaxIrrDur*	Maximum duration of an irrigation (h)	US
	37	*IrrPlus*	Additional irrigation water (%)	US
Soil hydraulic properties	38	*BD*	Bulk density (g cm^−3^)	(SS) – AS
	39	*FC*	Field capacity (m^3^ m^−3^)	(SS) – AS
	40	*WP*	Permanent wilting point (m^3^ m^−3^)	(SS) – AS
	41	*SAT*	Saturated water content (m^3^ m^−3^)	(SS) – AS
	42	*Ks*	Saturated hydraulic conductivity (cm d^−1^)	(SS) – AS
Plant growth	43	*Plts_DW*	DW of plantlets at transplanting (g)	US
	44	*Plts_Ref*	Reference dry weight of plantlets at transplanting (g	C
	45	*Plts_GR*	Initial growth rate (lag phase at plantlets stage) (g d^−1^)	C
	46	β_*1*_	β_*1*_ parameter of the logistic function for shoot growth	C
	47	β_2_	β_2_ parameter of the logistic function for shoot growth	C
	48	β_3_	β_3_ parameter of the logistic function for shoot growth	C
	49	*Root_r_ini*	Initial root radius (cm)	US
	50	*Root_r_max*	Maximum root radius of the most efficient part (cm)	C – L
	51	*Root_h_ini*	Initial root depth (cm)	US
	52	*Root_h_max*	Maximum root depth of the most efficient part (cm)	C – L
	53	*Root_d_max*	Number of days to reach maximum values (DAT)^b^	C
	54	*Hrvst_type*	Stage of the crop at harvest (full ripen fruit / immature fruit/ vegetative growth)	US
	55	*Tbase*	Base temperature (°C)	C – L
	56	*T_M1_*	Maximum temperature (°C)	C
	57	*T_M2_*	Cut-off temperature (°C)	C
	58	*Flw_beg*	Beginning of flowering (°Cd)	C
	59	*Flw_dur*	Duration of flowering (°Cd)	C
	60	*Flw_Tmax*	Maximum temperature for flowering period (°C)	C
	61	*Kts*	Averaged dry biomass response factor to water stress	C – L
	62	*K*_*T*1_	Shape coefficient for the adjustment of T_*M*2_	C
	63	*K*_*T*2_	Shape coefficient for the adjustment of T_*M*2_	C
	64	*K*_*T*3_	Shape coefficient for the adjustment of T_*M*2_	C
	65	*K*_*SDW*1_	Shape coefficient for the adjustment of the expected final SDW	C
	66	*K*_*SDW*2_	Shape coefficient for the adjustment of expected final SDW	C
ET_0_ assessments	67	*ET0_method*	Method for ET_0_ estimation [selection of: ET_0_ from the nearby meteorological station (S) / Penman-Monteith (PM) / Priestley-Taylor (PT) / Hargreaves-Samani (HS)]	US
	68	*E_*H*_*	Locally calibrated HS function coefficient	C – L
	69	*Albedo_PT*	Locally and crop calibrated albedo coefficient	C – L
	70	*Alpha_PT*	Locally calibrated PT function coefficient	C – L
	71–75	*HCmeteo1–5*	From 1 to 5 meteorological dataset files to be previously prepared and used only in case of Hargreaves calibration	(SS)
Nitrogen	76	*Pre_N*	Pre-planting N (kg ha^−1^)	US
	77	*IrrWt_N*	N in the irrigation water (ppm)	US
	78	*a*	Critical curve parameter	C – L
	79	*b*	Critical curve parameter	C – L
	80	*N_min_res_1*	Minimum N reserve in the soil (kg ha^−1^) in the initial phase	C
	81	*N_min_res_2*	Minimum N reserve in the soil (kg ha^−1^) in the mid phase	C
	82	*N_min_res_3*	Minimum N reserve in the soil (kg ha^−1^) in the final phase	C
	83	*N_year*	N applied in the same year on the same soil (kg ha^−1^)	US
Kc options	84	*KcMode*	Kc mode selection (single or dual)	US
	85	*T*_1_	Time in days to complete the initial phase (DAT)	C
	86	*T*_2_	Time in days to start the middle phase (DAT)	C
	87	*T*_3_	Time in days to complete the middle phase (DAT)	C
	88	*T*_4_	Time in days to complete the cycle (DAT)	C
	89	*Kc_ini*	Kc-valueduring the initial growth stage of the cyc	C – L
	90	*Kc_mid*	Kc-value during the middle growth stage	C – L
	91	*Kc_end*	Kc-value during the late growth stage	C – L
	92	*Kcb_ini*	Kcb-valueduring the initial growth stage of the cycle	C – L
	93	*Kcb_mid*	Kcb-value during the middle growth stage	C – L
	94	*Kcb_end*	Kcb-value during the late growth stage	C – L
	95	*SC_ini*	Soil covered during the initial growth stage of the cycle (%)	C
	96	*SC_mid*	Soil covered during the middle growth stage (%)	C
	97	*SC_end*	Soil covered during the late growth stage (%)	C
	98	*HP_ini*	Average plant height during the initial phase (cm)	C
	99	*HP_mid*	Average plant height during the mid-phase (cm)	C
	100	*HP_end*	Average plant height during the final phase (cm)	C
SOM mineralization	101	*I*	Mineralization weight fact	US
	102	*Ts*	Tillage factor	US
	103	*MPD*	Maximum plough depth (cm)	US
	104	*fr*	Crop residues management	US
Pedo-transfer functions	105	*Ped_met*	Pedotransfer method for estimating FC and WP from soil texture	US
Runoff	106	*RO_met*	Selection of the method (User defined / Curve Number)	US
	107	*Init_abst*	Initial abstraction (only in the case of an user defined approach)	US
	108	*Eff_rain*	Percentage of rain that is considered effective (only in the case of a user defined approach) (%)	US
	109	*CS_arr*	Crop/soil arrangement (in the case of the Curve Number method)	US
	110	*Hy_cond*	Hydrological soil conditions (in the case of the Curve Number method)	US
	111	*CR_pres*	Crop residues presence (in the case of the Curve Number method)	US

a SS, site-specific, parameters related to the site; US, user-specifc, parameters related to the crop and the soil management; C, parameters which need to be calibrated on each crop from specific research; L, parameters generally available from the literature; AS, parameters for which the value is automatically suggested by the software.

b DAT: days after transplant.

The form format has a menu bar on the left of the form designed so that users move logically from top to bottom along a taskbar, individually selecting appropriate pages within the form before moving to the next step. The order was determined by the logical sequence.

A maximum of 111 entries are requested to start a crop. However, excluding one that is automatically selected (AS) by the software (entry # 14: USDA soil classification), they can be distinguished in the following types: 53 are crop calibration parameters, which are automatically retrieved by the software after selecting the crop [35, derived from specific calibration from basic research into each crop (C), and 18 are generally available in the literature (L) (e.g., Kc-values, HI, Critical N curve parameters, RAW, base temperature)], and 57 are the real entries managed by the user [34 are user specific (US), and 23 are site specific (SS)] (Table 3).

The site-specific entries are mainly descriptive information or data already available to the user (e.g., geographical position, location and NVZ identifiers, soil physical and chemical characteristics, name of climate files, E_*H*_ Hargreaves function coefficient, α Priestley Taylor function coefficient). The user-specific entries are related to the crop management data which depends on user choices (e.g., planting date, plant distance, emitter flow rate, cv. name, meteofile, ET0 method, mulching, etc.). Among the SS entries, entry # 4 (*meteofile*) (Table 3), even if highly recommended, is not mandatory when no pre-season plan is performed (DSS working only in *Day to day in-season management* mode, see below), as well as the entries # 71 to # 75 (*HCmeteo1–5*) (Table 3) if ET_0_ is not calculated through the HS or PT functions or the EH coefficients for the HS function or α for the PT function have already been calibrated for the local conditions. The parameters related to soil hydraulic properties (entries # 38 to # 42 – Table 3) can be automatically selected by the software (AS-parameters estimated as a default setting as reported in the Section Physical and Hydraulic Soil Characteristics), however it is strongly suggested to use values from specific soil analysis data.

In DSS implementation as a web-based application, which is underway, a database repository of parameters for the calibrated crops (both C and L type parameters) will be available, so when a user selects a crop the set of calibrated parameters for that crop will be automatically retrieved by the software. The database of calibrated parameters will collect any crop calibrations that future research provides.

### Operational management of fertigation

**Figure 6:**
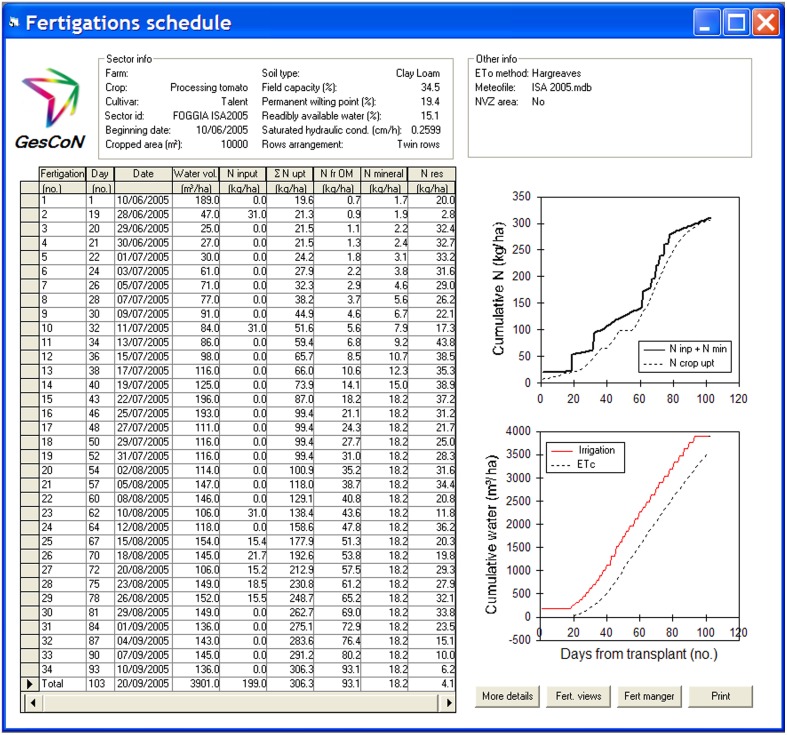
**Example of a final output of a fertigation management**. The output summarize in tabular form the fertigations suggested during the cultivation period, giving information on the suggested amount of water and N rate to apply at each single event, and other details on the estimated N crop uptake, N mineralization from SOM, interception of the mineral N reserve and the residual N in the soil. Some of the above data are also presented in graphical form in the two lateral graphs.

The main objective of *GesCoN* is to provide information to operationally assist the fertigation of a crop. In Figure 6 an example is given of a final fertigation schedule. The information provided by the DSS can be of two types and obtained through pre-season planning and/or day to day management.

#### Foresight pre-season plan and control for NVZ areas

Before the start of a crop season the program allows users to make a simulation of the crop growth giving a plan of the expected schedule of fertigations including dates, irrigation volumes and N rates as well as estimating the total N-amount that should be applied to the crop in order to obtain the expected yield. The simulation can only be performed if a dataset of multiannual averaged historical temperatures (maximum and minimum) data are available. As rainfall has an extremely heterogeneous distribution over time, the long-term mean data for rainfall is not used in the simulation. The foresight plan represents the first output of the program. If the farm is located in a NVZ area the foresight plan is necessary to forecast the N-requirement by the crop. If this is greater than the restrictions imposed by the law in these areas (170 kg ha^−1^ per year of maximum N inputs), the program advises that it is impossible to achieve the expected yield and recalculates a new maximum yield that can be feasible with the limited N-amount (*N_*nvz*_*) that it is possible to supply to the crop. In the calculation of the maximum potential SDW, under NVZ limitations (*SDW_*nvz*_*), the program takes into account the N availability in the soil root zone (either from SOM mineralization or from initial mineral N stock) and the N applied to the soil in previous crops during the same year.

When not in an NVZ area the pre-season plan can be considered optional, but it is however recommended as it gives the farmer the possibility of testing the DSS against normal climatic conditions for that site (DSS benchmarking on crop and site) and to evaluate the provisional plan for the fertigation season, which can be useful for planning all the operations and preparing irrigation equipment, water sources, and fertilizers.

#### Day to day in-season management

The real-time fertigation management is performed using the “*Day to day in-season management”* where the DSS requires the daily input of meteorological data. The maximum and minimum temperatures and rainfall (if any) are mandatory. If an ET_0_-value is not provided by a nearby meteorological station, additional data are requested depending on the method selected for the estimation of ET_0_: maximum and minimum relative humidity, wind speed, and solar radiation in the case of the PM method; solar radiation in the case of the PT method. On the contrary, no further input of climatic data is requested if the HS method is selected to estimate ET_0_.

On the basis of these data the DSS gives real-time information on growth, water consumption and N uptake of the crop. When a fertigation event is triggered, the DSS advises on the need to start irrigation or fertigation, giving all the information on the water irrigation volume and the N rate (if required) to be applied (see Supplementary Figure).

If a pre-season plan has also been made at the beginning of the crop, the farmer can evaluate the deviations of real-time growth, water consumption and N uptake compared from those forecasted under “normal” climatic conditions for that area.

When a fertigation is scheduled for a given day, its having been carried out must be confirmed by selecting the relative checkbox. If there are variations in the amount of N and/or water delivered to the crop, the correct amounts must be properly recorded in the specific input box.

### Meteorological files

To run a per-season plan, a file with a meteorological data set must be previously created for each specific location. The software requires a Microsoft Access file format with each record containing the multiannual mean of the daily climate data of the nearest weather station (*Meteofile*). The filename of this dataset represents the zone identifier and must be stored in the “Meteo” subfolder within the installation folder.

Further additional meteorological files from 1 to 5 (*HCmeteo1–5)* may be required if the HS or PT calibration options is selected. In this case each meteorological file must contain the climatic data of a single year and all the meteorological variables are mandatory, or alternatively only # 9.

In every case a climate file must be created in a tabular scheme with days in the rows (records) and meteorological variables in the columns (fields) and organized in following order:

1. Julian day, *JD*
2. Rainfall (mm), *Rain*
3. Minimum air temperature (°C), *Tmin*
4. Maximum air temperature (°C), *Tmax*
5. Minimum relative humidity (%), *RHmin*
6. Maximum relative humidity (%), *RHmax*
7. Wind speed (m s^−1^), *WS*
8. Solar radiation (MJ m^−2^ d^−1^), *SR*
9. Reference evapotranspiration (mm), ET_0_

Variables 3, 4 (for the *Meteofile*), and 2 (for day-by-day in-season management) are mandatory, while those from 5 to 9 are optional. If data for variables 5–8 are available the DSS can calculate, with its specific routine, ET_0_ using the PM model, otherwise the PT (if relative humidity, wind speed are missing) or the HS model (if only the *Tmin* and *Tmax* are available) will be used. As an additional option, if an ET_0_-value from the nearby station is available (variable 9) the DSS can use it as a reference evapotranspiration value.

## Conclusions

The DSS is intended as a tool that can be easily used by a technician or a farmer to manage both irrigation and N fertilization through fertigation for open field grown vegetables.

The crop growth model is based on thermal time using a simplified logistic model which requires only daily temperature (*Tmin* and *Tmax*) as input data, so reducing the need for off-farm inputs compared with the more detailed mechanistic models which describe growth from underlying physiological processes (e.g., photosynthesis and respiration) in relation to the environment. Despite the simplicity of the growth model, it takes into account water and thermal stresses and the *SDWcheck* procedure allows in-season calibration of the model through a dynamic adaptation of the growth rate to the specific genetic and environmental conditions.

N crop demand is quantified on the basis of single-plant nitrogen concentrations through the N critical approach, where the “critical” value corresponds to the minimum N concentration permitting maximal crop growth. N delivered from SOM mineralization and N depletion from the root volume by excessive rains are also estimated.

For the water balance estimation the only daily climatic data needed are two temperature values (*Tmin* and *Tmax*) and rainfall, all of which can be easily recorded on-farm. If the ET_0_-value is not provided by a nearby meteorological station and the PT or PM method is selected for estimating reference evapotranspiration other meteorological data are also needed, that are radiation, air humidity, and wind speed in case of PM, or only radiation in case of PM.

*GesCoN* offers the possibility of using the dual Kc approach for estimating crop evapotranspiration, considering the presence of soil mulching. Rain runoff and deep percolation are also estimated.

The DSS has some original approaches, such as the in-season calibration of the growth model, the routine for calibrating the HS and PT models to local conditions and the estimation of the real water amount supplied by wet bulbs to roots evaluating the effect of row type and plant distribution.

*GesCoN* operates in real-time during crop cultivation giving daily recommendations on water and N requirements to achieve the maximum potential yield for that crop in that area. It can also be run as a pre-season simulation using historical temperature data, making it possible to test the DSS against normal climatic conditions for that site (DSS benchmarking on crop and site) and to evaluate the provisional plan for the fertigation season, which can be useful for planning all the operations and preparing irrigation equipment, water sources and fertilizers. The pre-season plan is necessary when operating in EU designated nitrate vulnerable zones, providing the basis for the adaptive control of the maximum potential yield that can be achieved under the legal limitations in terms of N distribution and taking into account the potential N availability from SOM mineralization.

### Conflict of interest statement

The authors declare that the research was conducted in the absence of any commercial or financial relationships that could be construed as a potential conflict of interest.
