# Supplementary material for: A decision support system (GesCoN) for managing fertigation in open field vegetable crops. Part I—methodological approach and description of the software
Source: Front Plant Sci. 2015 May 20;6:319. doi: 10.3389/fpls.2015.00319 (PMC4438600; doi:10.3389/fpls.2015.00319)
Supplement: Supplementary file 1 [file Image1.PDF]

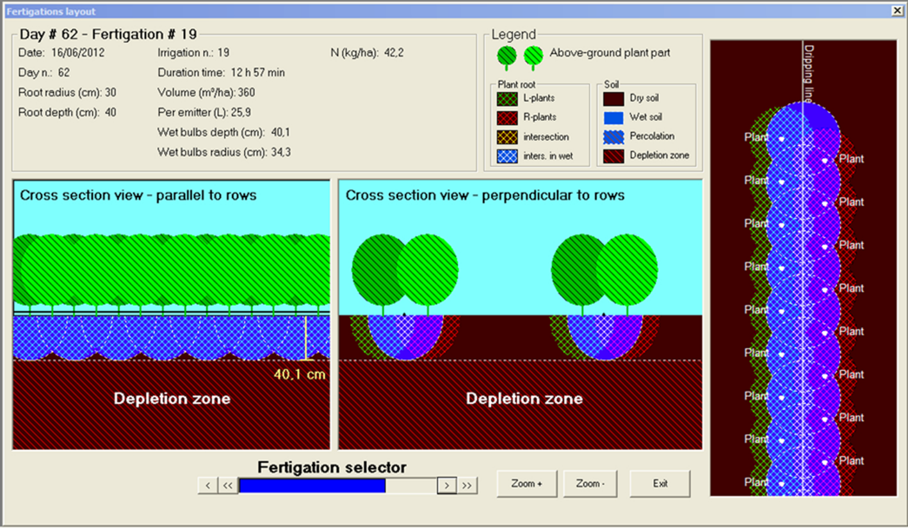

SUPPLEMENTARY IMAGE | Example of a layout provided by GesCoN for a fertigation event. It is possible to visualize from different points of view the simulation of the wetting front, of plant roots and of their interaction. The most relevant fertigation data, such as the specific fertigation duration, water volume and N rate to apply and the measures of the simulated wet bulbs and root apparatus are also provided.
